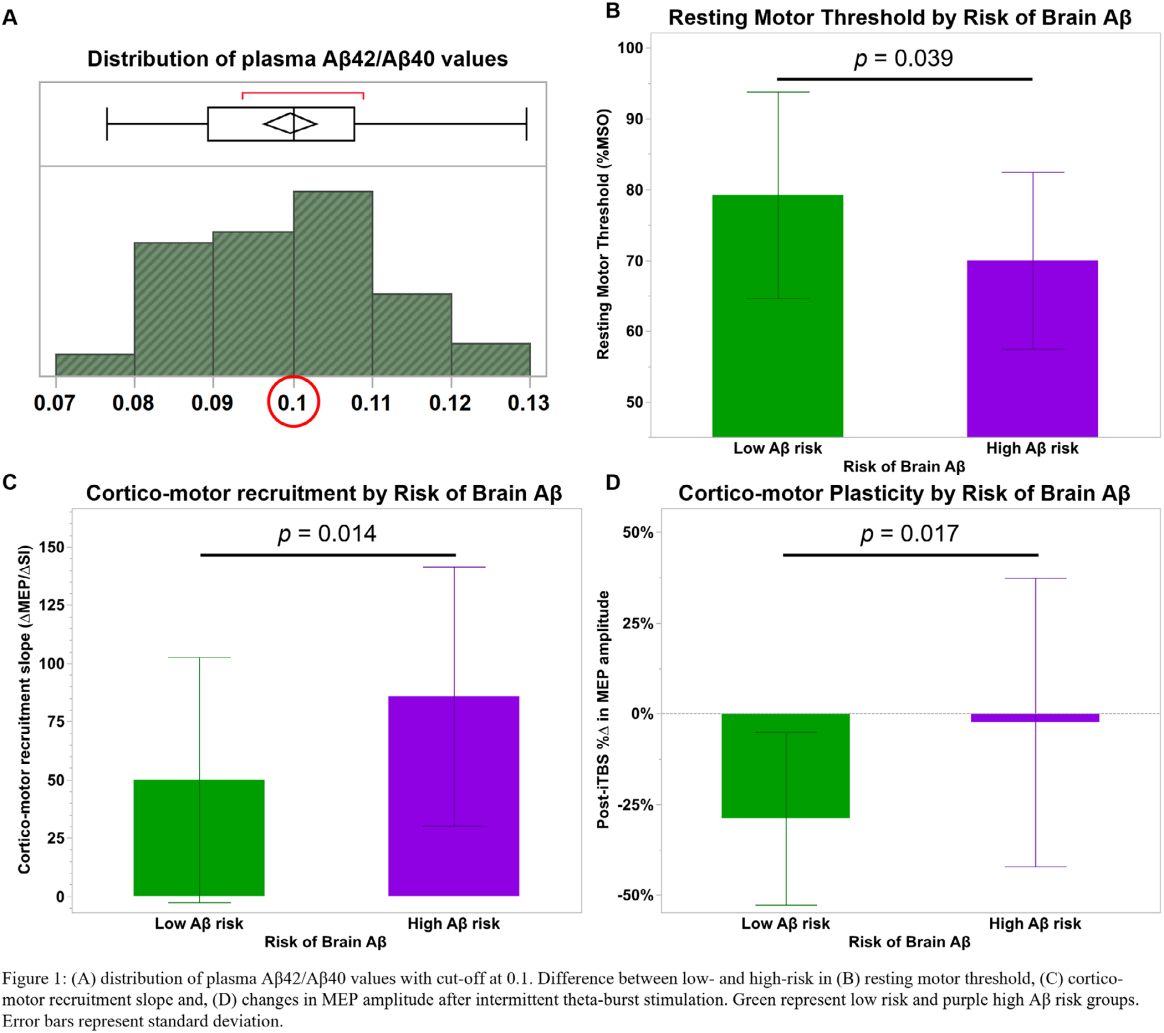# Alterations in cortical excitability and plasticity are associated with amyloid accumulation in cognitively unimpaired elderly

**DOI:** 10.1002/alz70856_105060

**Published:** 2026-01-07

**Authors:** Giacomo Bertazzoli, Brice Passera, Recep Ozdemir, Stephanie S. Buss, Mouhsin Shafi, Peter J. Fried

**Affiliations:** ^1^ Berenson‐Allen Center for Noninvasive Brain Stimulation, Beth Israel Deaconess Medical Center and Harvard Medical School, Boston, MA, USA; ^2^ Department of Neurology, Harvard Medical School, Boston, MA, USA; ^3^ Harvard, Boston, MA, USA

## Abstract

**Background:**

Alzheimer's disease (AD) is a progressive neurodegenerative disorder characterized by early amyloid‐beta (Aβ) accumulation, which can precede clinical symptoms by years. Plasma Aβ42/Aβ40 oligomers have emerged as a surrogate biomarker for brain amyloid, providing a non‐invasive method to identify individuals at high risk. Cortical excitability and long‐term potentiation (LTP)‐like plasticity, assessed via transcranial magnetic stimulation (TMS), are impaired in early symptomatic stages of AD. Aβ disrupts normal glutamatergic activity, increasing cortical excitability and reducing LTP‐like plasticity. This study tests the hypothesis that cortical excitability and plasticity are associated with plasma Aβ42/Aβ40 levels in cognitively unimpaired individuals to develop neurophysiological markers for early Aβ‐related dysfunction.

**Methods:**

Plasma Aβ42/Aβ40 levels were obtained in 58 cognitively normal adults (32 women, aged 58–89). MRI‐guided TMS assessments from the left motor cortex were performed in a subset. We used the Aβ42/Aβ40 cutoff of <0.1, based on Rissman et al. (2024; doi: 10.1002/alz.13542), to classify participants at high risk for brain amyloid. Cortical excitability was measured by resting motor threshold (RMT) and corticomotor recruitment slope (change in MEP amplitude between 120% and 135% RMT). LTP‐like plasticity was assessed by changes in excitability post‐intermittent theta‐burst stimulation (iTBS). Group differences were tested using non‐parametric Wilcoxon tests (α=0.05).

**Results:**

Data are reported in mean ± standard deviation. Of the 58 participants, 50% were classified as high Aβ risk (Figure 1A). The high‐risk group showed a lower RMT (70.0 ±12.5) than the low‐risk group (78.5 ±14.4), *p* = 0.039 (Figure 1B). The corticomotor recruitment slope was also greater in the high‐risk group (85.8 ±55.6) vs. low‐risk (50.0 ±52.3), *p* = 0.014 (Figure 1C). Both groups exhibited decreased MEP amplitude following iTBS, but the decrease was smaller in the high‐risk group (‐2.3 ±39.8%) vs. low‐risk (‐28.9 ±23.8%), *p* = 0.017 (Figure 1D).

**Conclusions:**

In cognitively unimpaired elderly, a higher risk of brain Aβ is associated with altered cortical excitability and plasticity, similar to changes in symptomatic AD. TMS may provide early markers of amyloid‐associated neurophysiological dysfunction.